# Culture’s Place in Quality of Care in a Resource-Constrained Health System: Comparison Between Three Malawi Districts

**DOI:** 10.1177/10497323211037636

**Published:** 2021-09-28

**Authors:** Patrick B. Patterson, Zubia Mumtaz, Ellen Chirwa, Janet Mambulasa, Fannie Kachale, Josephat Nyagero

**Affiliations:** 1 University of Alberta, Edmonton, Alberta, Canada; 2 University of Malawi, Zomba, Malawi; 3 Amref Health Africa, Nairobi, Kenya; 4 Government of Malawi, Lilongwe, Malawi

**Keywords:** care provision, organizational culture, quality improvement, Malawi, Organizational ethnography

## Abstract

Public health scholars describe “culture of quality” in terms of desired values, attitudes, and practices, but this literature rarely includes explicitly stated theories of culture formation. In this article, we apply Fredrik Barth’s transactional model to demonstrate how taking a theory-centered approach can help to identify what would be necessary to foster “cultures of quality” outlined in the public health literature. We draw on data from a study of the Republic of Malawi’s Performance and Quality Improvement for Reproductive Health initiative. These data were generated in 2017–2018 through a 6-month organizational ethnography in three facilities selected to represent a range of districts with differing social and economic contexts. Our analysis revealed facility-level organizational cultures in which staff valued providing care, but responded to structural constraints by normalizing divergence from quality-of-care protocols. These findings indicate that sustaining a quality-oriented organizational culture requires addressing underlying conditions that generate routine experiences and practices.

The Republic of Malawi has attempted to improve quality of maternal health care, including the national Performance and Quality Improvement for Reproductive Health (PQI-RH) initiative launched in 2006 (Rawlins et al., 2013). Malawi’s national maternal mortality rate remains high, however, at 439/100,000 live births (National Statistical Office, 2017). Quality of care has both biomedical and non-biomedical aspects, and while evidence-based approaches emphasize scientifically validated clinical practices (Raven et al., 2012), public health scholars recognize that sociocultural factors also play a role (Bouchet et al., 2002; Brown, 1995; Raven et al., 2011). Brown goes so far as to describe “culture of quality” as “the most intangible and arguably the most important element of a QA [Quality Assurance] program” (Brown, 1995, p. 420), and organizational culture may be a factor in outcomes from Malawi’s quality improvement efforts.

## Organizational Culture in Public Health

While changing organizational cultures could be important for improving quality of care (Bouchet et al., 2002; Brown, 1995; Heiby, 2014; Øvretveit, 2002; Raven et al., 2011; Umar et al., 2009; Whittaker, 1999), there is little agreement in the public health literature about what cultures are, or the feasibility of intentionally changing them (CFIR Research Team, 2021; Scott et al., 2003). Scott et al. (2003) identify two main ways the concept has been used in public health. In the first, culture is seen as an attribute of the organization (Scott et al., 2003). Work following that approach has focused on description of occupational cultures’ features. Theories about how cultural elements originate follow a management model that emphasizes leadership and training to promote favored cultural practices and attitudes (Brown, 1995; Curry et al., 2011, 2018; Heiby, 2014; Raven et al., 2011; Whittaker, 1999). The second approach treats culture as a system which is involved in all aspects of organizational life and is difficult to modify (Scott et al., 2003). Although some researchers consider structural or contextual factors (CFIR Research Team, 2021; Curry et al., 2018; Dutta & Basu, 2008; Dutta & Dutta, 2012), both approaches treat culture as primarily a psychological or ideological phenomenon, equating it with attitudes and values (CFIR Research Team, 2021; Scott et al., 2003). Both approaches assume that culture causes behavior, and poor evidence-based practices stem from failure to establish quality-focused values and attitudes (Bouchet et al., 2002; Brown, 1995; Curry et al., 2011, 2018; Raven et al., 2011; Whittaker, 1999). The first approach treats culture as an independent variable that can be deliberately modified (Scott et al., 2003). When treated as a variable, organizational culture is easily included in evaluations and the first approach can be considered the dominant perspective in public health.

Assumptions embedded in the approaches to organizational culture have consequences for policy and practice. Scott et al. (2003) point out that to change culture effectively, it is necessary to understand its nature, how it is sustained, how change processes occur, and how the culture fits within the wider context. Several of those elements fall outside what is usually considered in management-oriented models of culture, but they are exactly what anthropological and sociological theories were developed to examine (Douglas, 1982) and concepts from those fields could strengthen approaches used in public health. Scott et al. (2003) “treat culture as an emergent property, concomitant with its status as a social institution . . . [and] assume that its main characteristics can at least be described and assessed” (p. 112). Although there is no dominant theory of culture formation and change among anthropologists or sociologists, the transactional model developed by Fredrik Barth to study culture formation and change (Barth, 1966, 1989, 1993) takes a similar perspective to Scott et al. (2003) and is suited to analysis of the points they set out.

## Barth’s Transactional Model of Culture

The central premise in Barth’s transactional model is that cultures are patterns of behavior and thought generated through repeated social actions in specific settings (Barth, 1966, 1989, 1993; Jenkins, 2008). Material and social features of contexts act as constraints or incentives on actions, making some behavioral strategies more consistently satisfying than others for the individuals involved (Barth, 1966). People evaluate the outcomes of their interactions and adjust their behaviors and assumptions. Over time, people facing a consistent set of constraints tend toward using similar preferred behaviors and ideas, which become institutionalized as the social group’s culture: behavioral norms, values, and assumptions (Barth, 1966; Jenkins, 2008). Once established, cultural concepts and norms act as an additional layer of constraints or incentives group members face and reinforce the tendency to use particular strategies (Barth, 1966; Jenkins, 2008). Within Barth’s model, cultural change occurs when aspects of the surrounding context are altered in ways that constitute new constraints or incentives for action by individuals (Barth, 1966, 1989). The model assumes that people are intelligent agents who are capable of innovation. Therefore, cultural change may also occur when individuals apply a new behavior or idea that proves effective during interactions and becomes widely adopted (Barth, 1963, 1966).

In this article, we apply Barth’s transactional model to analysis of ethnographic data generated in 2017–2018 in Malawi at three publicly funded facilities that implemented the PQI-RH initiative. Our goal is not to promote the transactional model as *the* way to analyze culture in public health; Dutta has successfully applied Giddens’ Structuration Theory (Dutta & Basu, 2008; Dutta & Dutta, 2012; Sun & Dutta, 2016) and other theories are appropriate for the task. Rather, we demonstrate how placing an explicitly stated theory of culture formation and change at the center of analysis helps to identify what would be necessary to foster organizational cultures closer to the hypothetical “culture of quality” outlined in the public health literature (Bouchet et al., 2002; Brown, 1995; Heiby, 2014; Øvretveit, 2002; Raven et al., 2011; Umar et al., 2009; Whittaker, 1999).

## Health Care in the Republic of Malawi

Before turning to analysis of facility-level processes, it is important to have a general understanding of the wider context the facilities operated within during our 2017–2018 ethnographic fieldwork. The government of Malawi had limited financial resources available to fund health services. The country’s per capita income was low, due to dependence on agriculture for both the household-level subsistence and commercial exports (World Bank, 2019). Tax revenues were also modest because much of Malawi’s economic activity occurred within the informal sector (Government of Malawi, 2017). The country’s publicly funded health services relied on international aid for up to 65% of the annual budget (Mbuya-Brown & Sapuwa, 2015; Ochalek et al., 2018; Wilhelm et al., 2016), then, in 2014/2015, funders withdrew budgetary support following a corruption scandal (Adhikari et al., 2019; Manda-Taylor et al., 2017; Mbuya-Brown & Sapuwa, 2015). Over the period between 2013 and 2017, Malawi’s currency, the Malawi Kwacha (MWK), was also devalued by 50% compared to the US dollar (ExchangeRates.org.uk, 2018a, 2018b), making imported medical supplies more expensive. Malawi’s infrastructure could also present obstacles to health care provision. Electricity shortages in the national power system were common (Kaunda & Mtalo, 2013; Suhlrie et al., 2018). Blackouts made district-level water systems that relied on electrical pumps unreliable and contributed to communications and data-handling failures.

Politically, Malawi had been a stable multiparty republic since 1993. The country was administratively split between the Northern, Central, and Southern Regions and was divided into 28 districts empowered to carry out local governance. Governmental authority was centralized, with senior officials controlling key resources. The Ministry of Finance allocated Other Recurring Transactions (ORT) funds to each district, which were used to pay for non-salary and non-medical supply expenses (Manda-Taylor et al., 2017). The national Health Service Commission (HSC) recruited and hired Ministry of Health (MoH) personnel and paid their salaries (Africa Health Workforce Observatory, 2009; Government of Malawi, 2018). Approval of requests from districts to hire and deploy new staff could be delayed, and understaffing was common (Africa Health Workforce Observatory, 2009; Chimwaza et al., 2014). Medical supply systems were also centralized in two trust organizations. Central Medical Stores Trust (CMST) procured and distributed pharmaceuticals and medical supplies (Central Medical Stores Trust, 2018). It was the sole authorized supplier to MoH facilities, which requisitioned supplies through a debit system. Malawi Blood Transfusion Services (MBTS) provided hospitals with safe supplies of blood and blood products (Kongnyuy & van den Broek, 2007; Malawi Blood Transfusion Service, 2019). The government was in an ongoing process of decentralizing decision-making (Tambulasi, 2009) and at the time of this study some authority over budgets and public service hiring was being transferred to District Councils.

The Ministry of Health was internally organized into three levels: headquarters, which set overall policies; zones, which oversaw several districts; and districts, which provided most services (Ministry of Health and Population, 2019a). District Health Management Teams (DHMTs) managed district-level health services. Each DHMT was headed by a District Health Officer (DHO), who was supported by a District Medical Officer (DMO) to manage clinic staff, a District Nursing Officer (DNO) to manage nursing staff, and several non-medical administrators. Malawi’s four central hospitals reported directly to MoH headquarters rather than to district officials. District-level facilities included health centers and district hospitals (Ministry of Health and Population, 2019b). Health centers provided basic emergency obstetric and newborn care (EmONC), which included administering drugs to support delivery, manual removal of placenta and retained products of birth, assisted vaginal delivery, and basic neonatal resuscitation (. District hospitals provided comprehensive EmONC, including surgical procedures, such as caesarian section and blood transfusion (World Health Organization et al., 2009). Medical doctors were in short supply in the MoH and most clinical care was provided by clinical officers and clinical technicians, para-medical staff holding 4-year medical degrees or 3-year diplomas, respectively (Jiskoot, 2008; van Amelsfoort et al., 2010), or nurse-midwives. To promote facility births, maternity wards in district hospitals and some health centers also functioned as waiting homes, with expectant mothers admitted up to 1 month prior to their estimated delivery dates (Singh et al., 2018; Suwedi-Kapesa & Nyondo-Mipando, 2018).

Malawi’s PQI-RH initiative was implemented at most facilities that provided basic or comprehensive EmONC services (Rawlins et al., 2013). The Ministry of Health and Jhpiego, a US-based non-profit health organization, developed the initiative based on Jhpiego’s Standards-Based Management and Recognition approach (Necochea et al., 2015; Rawlins et al., 2013). The MoH worked with Jhpiego to create evidence-based standards for maternal care. The DHMT in each participating district then established quality improvement teams, whose members were trained to evaluate compliance with the national standards, identify gaps in care, plan ways to address the gaps, and work with managers and staff to carry out activities to meet quality-of-care targets. Quality improvement teams would then evaluate the progress and use that data to work with care providers to adjust practices. Facilities could request an external evaluation, and if they were successful, their achievement would be recognized with an award (Rawlins et al., 2013).

## Method

In this article, we draw on data from a multimethod study of Malawi’s PQI-RH initiative. Data were generated in three modules: Module 1 included in-depth interviews with policy makers and implementers, and a review of policy-related documents available in the public domain; Module 2 was a quantitative quality of care survey at six facilities implementing the PQI-RH initiative; Module 3 was a 6-month organizational ethnography focused on a subset of three facilities surveyed in Module 2. Data used in this article were generated only in Module 3, the organizational ethnography. Facilities studied in Module 3 were purposively selected (Robson & McCartan, 2016) to include sites that varied in PQI-RH implementation success and in their social and economic contexts. Characteristics of the sites are shown in Table 1.

**Table 1.** Site Characteristics and Resource Availability.

Facility characteristics	Site A	Site B	Site C
District/Context	Rural, Central Region - Inland - Approx. 200,000 pop. - Farming	Rural, Central Region - Lakeside - Approx. 280,000 pop. - Farming, fishing, tourism, commercial agriculture	Urban, Southern Region - Inland - Approx. 750,000 pop. - Services, farming, tourism
PQI-RH Targets achieved	No	Yes	Yes
Facility type	District hospital	District hospital	Health center
Facility total capacity	180 beds	400 beds	18 beds
EmONC Services	Comprehensive	Comprehensive	Basic^a^
Approx. ORT funding (monthly)	MWK 14 million/US$19,300	MWK 22 million/US$30,000	MWK 40 million/US$55,000
Facility medical staff	2 MDs 8 Clinical Officers/Techs 30 Nurse-Midwives	2 MDs 10 Clinical Officers/Techs 30 Nurse-Midwives	0 MDs 2 Clinical Officers/Techs 12 Nurse-Midwives
Functioning ambulances	2	2	4
Electricity	Intermittent	Reliable	Intermittent
Water supply	Piped; intermittent	Piped; intermittent	Piped; reliable
Medical supplies	Stockouts common	Stockouts common	Stockouts common
Blood supplies	Stockouts common	Stockouts common	NA; transfusions not performed

*Note.* PQI-RH = Performance and Quality Improvement for Reproductive Health; EmONC = emergency obstetric and newborn care; ORT = Other Recurring Transactions.

a No District Hospital; patients requiring comprehensive EmONC referred directly to Central Hospital.

Ethnographic data were generated through observation of care provision and semi-structured interviews (Atkinson et al., 2001; Leslie et al., 2014). Observations focused on conditions in facilities and their maternal care wards, actions care providers took, interactions between care providers, and interactions between care providers and clients. An observation guide was not used. Instead, team members were trained to watch for a general set of actions and were assigned each day to specific facility areas to follow opportunities as they emerged. While conducting observations, team members asked individuals to participate in semi-structured interviews. Interview participants (*n* = 121) included facility managers and administrators (*n* = 16), care providers (*n* = 28), support staff (*n* = 18), and patients or their family members (*n* = 59).

During the first 2 months of the ethnographic field study, two non-Malawian team members participated in data generation while training two Malawians in ethnographic research methods. For the remaining 4 months, the two Malawian researchers generated data. Members of the research team were not employed at any of the facilities and presented themselves as visiting researchers. Both Malawian field researchers were qualified nurses who had previously worked in the health system. They had also visited the facilities while conducting surveys in Module 2 and met some managers and care providers before starting the ethnographic observations.

Most interviews with MoH personnel were conducted in English. The Malawian researchers were fluent in Chichewa, a widely spoken language in Malawi, and conducted interviews with participants who did not speak English. Interviews conducted in Chichewa were translated into English and all interviews were fully transcribed. Transcripts and field notes were uploaded to NVivo 12 software (QSR International, n.d.) for data handling. The project received ethical approval from Malawi’s College of Medicine Research Ethics Committee (COMREC) and the University of Alberta, Canada. District-level managers were informed about the project before data generation began at sites they were responsible for, and individual participants were asked for informed consent prior to interviews.

Coding and data analysis proceeded in stages. Initially, descriptive open coding was used (Corbin & Strauss, 2008), which generated six general topic domains: quality improvement practices; patient care practices; management practices; health system resources; nongovernmental organization (NGO) partners and programs; and patient behaviors. Data within these domains were progressively divided into descriptive subcategories, which revealed a number of organizational structures, such as the MoH position within the Malawi government, formal MoH organization/hierarchy, and facility-level organization. Categories also described patient-care practices, behavioral norms, and explanations participants gave about their actions and circumstances they faced.

**Figure 1.**
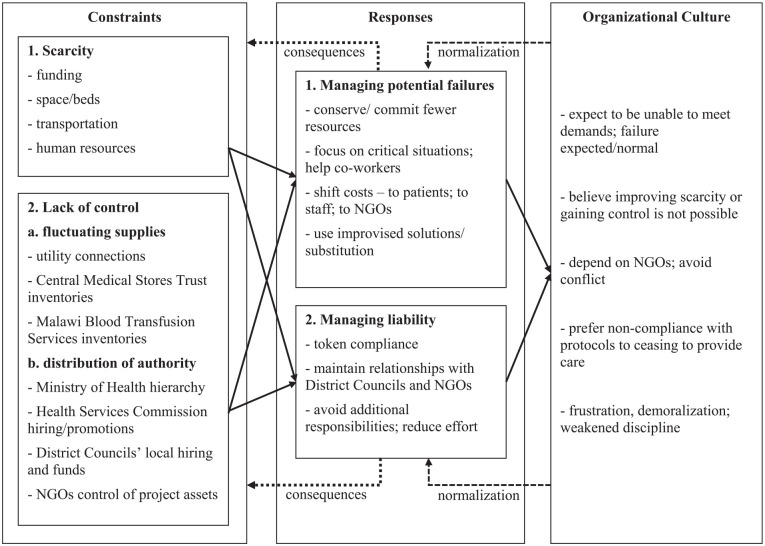
Links between constraints, responses, and organizational culture. *Note.* NGOs = nongovernmental organizations.

Based on findings from the descriptive open coding, the research team chose to carry out detailed analysis of organizational culture in the facilities. The second analytical stage used transactional theory (Barth, 1966, 1989, 1993) as a heuristic device to develop explanations for behaviors identified through the descriptive open coding. The analysis linked constraints at the facilities with specific behaviors and justifications participants used, which were taken to constitute elements of organizational culture. Linkages between constraints, responses, and cultural elements are shown in Figure 1. The analysis noted variations between the sites, including differences in norms and expressed values.

## Findings

Our analysis revealed facility-level organizational cultures in which behaviors, attitudes, and values revolved around coping with resource scarcity and lack of control. Although the constraints MoH personnel faced, their responses to those circumstances, and the features of the organizational cultures are interconnected within dynamic processes, we describe each separately in the following sections.

### Constraints on Managers and Care Providers

#### Scarcity

MoH managers and staff identified several causes of scarcity. Participants told us that the allocation of health care facilities and the funding to operate and maintain them were based mainly on the number of residents in each district. A manager at Site A explained,

they find that [population] equivalent to the budget that we have, which is a mistake . . . if you look at the population which we are serving, it is not just people in [this District], there are a lot from [neighboring District], because they don’t have a district hospital close to them.

Although the Ministry of Finance provided funding on a reliable schedule, participants often commented that the amount provided was not sufficient to meet basic operational needs. For example, a manager at Site B said, “we work based on the [ORT funding] figure they have given us . . . we have to sit down and bang our heads, how do we sustain [operations] on the [amount received]?.” Participants in all three districts identified numerous budget shortfalls.

Space in maternity and labor wards was also in short supply at the facilities. Site A and B’s maternity wards frequently had too few beds for expectant mothers, due to the hospital waiting home policy and high demand for facility delivery services. For example, during an observation period at Site A, the maternity ward had 40 beds and a nurse reported that there were 117 expectant mothers that day. Although Site C did not function as a prenatal waiting home, it also sometimes had too few beds to meet demand. Labor rooms were similarly crowded at all three facilities. For example, at Site B,

a patient was brought in from gynae [ward]; she was distressed. The assessment from gynae indicated that she was [dilated] 7 cm. She was given a bed; however, there was another patient who was on the bench waiting for a bed. She was not given a bed straightaway because they were short of beds and the beds were full. (Field Notes)

We observed similar crowding in the other facilities and there were often more women in labor than delivery beds.

Each of the districts covered large areas that included 10 to 20 health centers, in addition to the study sites, and participants identified transportation shortages stemming from competing demands. Ambulances transferred patients between facilities or to central hospitals if they required services that were not locally available. The district where Site C was located had no district hospital and staff at Site C referred patients requiring comprehensive EmONC directly to the city’s central hospital. Ambulances were also used for transporting supplies and taking managers to health centers for supervisory visits. We were told that the MoH had eight to 12 ambulances in each district, but most were “grounded” due to mechanical failures and lack of fuel.

The Site A and B facilities were also understaffed. In all three districts, DHOs and DMOs were the only MoH medical doctors and we observed that at Site A and Site B comprehensive EmONC procedures were usually performed by clinical officers or clinical technicians. Nursing position vacancy rates were high. A manager at Site A said, “here at the district hospital, we require two hundred nurses but we only have thirty-three.” Participants said that nursing departments in Site A and Site B’s districts had between 60% and 70% of positions vacant. Both districts employed approximately 60 nurse-midwives, with half assigned to the hospital and the remainder in health centers.

Site C’s district had a similar overall staff vacancy rate, but the situation differed because patients requiring comprehensive EmONC were transferred to the city’s central hospital. That district’s DHO and DMO rarely provided direct patient care, and there was less need for clinical officers. The Site C health center had a near-full staff complement, with several clinical technicians and approximately 12 full-time nurses. DHMT members told us Site C was not representative of the district’s facilities. A manager explained, “[in the] the new establishment each health center must have at least 16 midwives . . . we are far from achieving that.” Participants said most health centers in that district had only two or three nurse-midwives, fewer than needed to meet care demands.

#### Lack of control

While some necessities were continuously scarce, the availability of others fluctuated. As Table 1 indicates, we observed that electricity and water services were intermittent at some facilities. Supply chain disruptions also caused sporadic shortages. Facilities obtained specific materials through three distinct systems. We observed stock-outs in each system, but participants told us they occurred for different reasons. Procurement officers used ORT funds to purchase non-medical supplies from private companies, and participants attributed those stock-outs to having insufficient money available. A manager at Site B explained, “we don’t meet the needs that the user departments have, because of the shortages of funds . . . That’s why we are found with a lot of credits [debt]. We still owe many suppliers.” Pharmacy departments could only obtain medical supplies from CMST. Participants attributed medical stock-outs mainly to CMST having low inventories. A DHMT member at Site B said, “when any commodity is out of stock there, we have nothing to do. We just keep our fingers crossed.” Several pharmacy technicians explained that CMST often did not completely fill orders, and some items were unavailable for long periods of time. Centralized MBTS blood banks provided blood and blood products to Sites A and B, but participants told us that MBTS often had too few units in stock to fill requisitions. Blood shortages worsened at Site A during blackouts, due to spoilage when refrigerators failed.

Managers and staff at the facilities sometimes faced administrative barriers. The MoH formally defined the roles, responsibilities, and actions that personnel at each level could take. One manager at Site A explained that

[departmental supervisors] sit down and discuss quality issues in their departments and work out solutions. For the whole hospital, we have the hospital management team and, for the district, we have the district health management team . . . From there we report to the zone and the zone connects with the national office.

Managers had few transportation assets available to make supervision visits, and communication systems were sometimes unreliable, which delayed approvals from authorities.

The facilities’ managers and care providers also depended on other government branches and external organizations. Funding was determined by the Ministry of Finance, and the Health Services Commission was responsible for hiring new medical staff. Participants told us that from 2014 to 2016 new hiring and promotion was curtailed due to budgetary constraints, leaving many unfilled positions. Participants said that District Councils had authority to levy local taxes and fees and were gaining authority to hire “junior cadres”—health service employees who required little medical training. However, they told us that District Council members did not necessarily prioritize health. One explained,

it is up to me to say, “I need this, I need that.” However, you know, most of the council members are politicians . . . there are issues of bridges, housing blocks. They want to do that, so everyone can see what they have done.

Participants at Sites A, B, and C also frequently worked closely with non-NGOs that provided money and supplies. They said that NGO partners did not usually provide funds directly and instead purchased supplies on behalf of districts or provided supplies or equipment related to specific projects. Supplies and project-oriented money provided at the district level were considerable; a DHMT member at Site C estimated that 80% of that district’s health sector resources came from NGOs.

### Responses to Constraints

#### Managing potential failures

Faced with scarcity, and lacking control over resources and systems they relied upon, MoH personnel at the facilities attempted to mitigate potential failures in several ways. One was by conserving money and supplies that would be difficult to replace. We were told that DHMTs stretched funding by purchasing large stocks of inexpensive supplies. For example, a food services manager at one facility explained,

previously, we were able to feed them [patients] all the six nutritional groups, but these days with our funding, it’s very difficult . . . We give [maize] porridge for breakfast, then at lunch hour we give them *nsima* [maize dumplings] with beans.

She specified that each patient received a total of 200 g of maize flour and 50 g of beans in the daily meals. Pharmacy technicians described using a similar strategy for requisitions, ordering medical supplies to fit within the budget, rather than what the facility needed. Care providers at Sites A and B also rationed blood products.

Managers directed scarce material and human resources to high-priority activities, including maternal care. DHMTs prioritized the use of ambulances for patient transfers, rather than for supervisory visits to facilities, and directed medical supplies to essential services. For example, a participant working in a facility pharmacy told us,

[when] gloves are running out, we stop giving them to the other departments and we keep them for the maternity ward because they need them more . . . [the facility in-charge] told me that when the rest of the departments come for gloves, we should not provide them.

Managers rationed electricity and water usage, allocating electricity from portable generators and solar panels to operating theater lights, neonatal incubators, and oxygen concentrators, while water was reserved for cleaning surgical instruments and patient care areas.

Maternal care also had priority for medical and nursing personnel. Managers in all three facilities attempted to cover vacant positions by assigning additional shifts to nurses and clinical personnel, who received locum payments for the additional hours they worked. Clinical officers often filled several positions simultaneously “on call,” and staff were assigned roles in addition to their routine duties, such as serving as quality improvement coordinators. In all three districts, staff responded to heavy workloads by focusing on the most urgent tasks, such as observing mothers presenting with signs of complications. Staff also supported each other. We noted examples where nurses or clinical officers filled in for a colleague when an individual was away from their station, or worked together when there was too much to do alone. A nurse at Site C told us, “[nurses in other wards] usually go back to maternity and help each other because the people in maternity are the ones who are there 24-hours and at night the maternity staff handle the outpatient patients.” Mutual support was especially common at Site C because the large staff allotment allowed nurses assigned elsewhere to routinely help in the maternity wing during breaks, or when no patients were at their assigned stations.

Managers attempted to meet operational requirements by shifting expenditures between budget categories, such as using ORT funds to make locum payments when staff worked extra shifts, which shifted staffing costs to the district budget. While some services, such as outpatient clinics, stopped providing services when medical stocks or personnel were unavailable, other activities, such as providing ambulance service, could not be curtailed when supplies ran out, or equipment failed. A DHMT member at Site B explained that when cost over-runs occurred, they “manage by borrowing . . . For example, in fuel, say its 3 million [MWK] per month but it’s not enough, so we borrow from the filling stations. They give us a limit that we can borrow to this amount.” Participants in all three districts told us that DHMTs amassed large debts.

Although procurement officers could seek alternative suppliers for non-medical supplies, DHMTs could not use ORT funds to buy medical stocks. Instead, managers and staff used resources from outside the government systems. Procurement rules discouraged staff members from paying out-of-pocket on behalf of patients, but participants described occasional examples, such as hiring taxis to transport patients when ambulances were unavailable. More often, districts shifted costs to patients, or their families. All inpatients were advised to bring a “guardian” to stay with them at the hospital to give personal care. Guardians provided much of the food patients ate, and reliance on them was normalized to the extent that communities constructed “guardians’ shelters” near Sites A and B to provide basic accommodations. Expectant mothers were also instructed to bring basic birthing supplies, such as wash basins, razor blades, wrapping cloths, and a plastic sheet. Having patients bring those supplies helped avoid stock-outs resulting from dependence on CMST. The health system also shifted transportation costs to patients. One manager at Site C explained, “as part of the antenatal training before a woman has her baby, they are told to save some money for a taxi just in case they will need to be referred [to the central hospital].” NGOs were another important external source of supplies. NGOs provided fuel for Site C’s portable generator, were installing solar panels and storage batteries at Sites A and C, and were digging a well and installing solar pumps and a storage tank at Site A to alleviate water shortages.

Managers and staff in all three sites routinely improvised solutions to cope with supply shortages, infrastructure failures, and understaffing. For example, when blood products from MBTS were unavailable, we observed that care providers at Sites A and B collected blood from volunteer donors for immediate transfusion, and participants said that during antenatal care, expectant mothers were advised to choose a guardian with a compatible blood type. Staff also substituted available materials for out-of-stock items. For example, staff at Site B used tubing and surgical gloves in place of urinary catheters and urine bags. Managers at Site B converted a waiting area into an improvised delivery room to address labor bed shortages. At Sites A and C, staff placed laboring women on spare mattresses on the labor room floor and all three facilities kept a supply of spare mattresses to accommodate maternity ward patients on floors. They also relied on substitute electricity or water supplies. Sites A and C used portable generators to power equipment, while staff used hand-held flashlights, or mobile phones for light during blackouts. Despite the increased risk of fires, staff at Site C also used candles to provide light when there was no fuel to run the portable generator. Patients who required supplemental oxygen were transferred to the central hospital. At Sites A and B, managers sent ambulances to bring barrels of water from wells to the facilities when piped water supplies failed.

Managers used a similar strategy to deal with personnel shortages. District Councils could hire workers who had little formal training, and DHMTs shifted tasks to such workers in understaffed facilities. For example, hospital attendants with no medical training worked in pharmacy dispensaries and sometimes distributed medications without supervision. Managers also shifted care provision to clinical interns, nursing students, and unemployed health degree graduates. Sites A and B hosted practicum placements and internships, where trainees provided care under the supervision of qualified staff. When nurses and clinical officers were overloaded or absent, students and interns worked unsupervised in the maternity wing. Nursing students were assigned to the Site C health center, but the facility had enough nurses that students did not work without supervision. Managers at Sites A and B also assigned shifts to individuals who had completed their clinical or nursing degrees, but were not yet hired through the HSC. A DHMT member in Site A’s district explained, “we are short-staffed, so we get those that are finished their college and have written their licensing exams, but they don’t have a job.” These workers were paid monthly allowances of 20,000 MWK (approximately $US 27.50) from ORT funding.

#### Managing liability for failures

Ministry of Health managers and supervisors sometimes followed policies because they were required to, rather than from commitment. For example, when discussing a maternal death audit, one participant said, “we were doing it because the zone [manager] will need to come and check those boxes again, so they should find that, at least, we have already filled it in.” Managers in that district treated the audit process as a reporting requirement, rather than as an opportunity to improve. Clinical staff and nurses deferred to supervisors when they were present and were reluctant to exercise judgment. A supervisor at Site B was critical of that attitude and explained, “when we [supervisors] sit in our chairs, they can’t make the decisions without us. They will need to ask, no matter how urgent the decision is.” Senior managers’ focus on assigning responsibility for mistakes could also contribute to poor coordination. A supervisor explained that, “we are working in parallel . . . my achievements, I want them to be mine, and his failure to be his.” In situations where patient care failures occurred, staff members were concerned with deflecting blame.

The DHMT members in Site C’s district acknowledged problems stemming from focusing on mistakes and instead used a management style that encouraged subordinates’ input. One explained that he tried,

to create a very good working relationship with the department heads and the in-charges of the respective facilities, because they are the ones on the ground . . . you can dictate, but you also have to ask, because when you dictate and then things go wrong, people will just look at you without helping you find solutions.

Staff at Site C described feeling able to discuss issues with their co-workers and supervisors, while the DHO in Site C’s district expressed confidence in their ability to implement changes.

Decentralization also placed DHMT members in the position of having to meet MoH superiors’ expectations while maintaining working relationships with multiple external parties. A DHMT member in Site C’s district explained that when working with outside organizations, “if we cannot deal with the problem immediately, we just give each other tasks to take back to our various secretariats . . . not that it will all be perfect, but it will be very conducive for both them and us.” District managers’ sense of vulnerability influenced how they interacted with NGOs. A DHMT member in Site B’s district used the analogy of a poor family to explain the relationship:

I am poor. I can’t even afford to buy a shirt for my child . . . somebody comes from elsewhere and says, “you know, I have brought shorts for your child,” when your priority is to buy the shirt. You cannot refuse that.

Participants said their districts accepted what NGOs offered, even when programs did not address their principal concerns.

Patient care personnel responded to intense workloads and uncertainty by reducing their efforts. That was sometimes a deliberate strategy. For example, we heard a staff member tell a colleague he was going to “assess a good number of mothers and then stop” because there were many patients and he was working alone. The colleague responded that he had previously done the same thing (Field Notes, Site A). In other cases, decreased effort stemmed from coming to work while unable to function. For example, “[two nurses] were discussing dealing with the on-duty clinical technician, and apparently he was having some mental health issue . . . He wasn’t doing anything; not seeing the patients, he was just sitting there. Things were getting backed up” (Field notes, Site C). Although these workers were present at the facilities, they did not contribute fully to patient care. Reducing effort increased pressure on colleagues and could become a source of conflict, as indicated by a nurse who

started complaining about her friend who didn’t come for duty . . . I asked her what happens when someone has not come for work, and she said, “nothing, it will just end like that.” Then she said she will still pay him back when she is on duty with him. She said she will deliberately not come to work. (Field Notes, Site B)

These disputes between care providers could undermine teamwork within the facilities.

### Occupational Cultures in Ministry of Health Facilities

Participants at all three sites told us that they valued serving their communities through the MoH. A nurse at Site C explained, “joining the government was not to get money. I wanted to be part of nursing, to save people.” Examples of paying out-of-pocket for supplies, and working very long shifts, suggest staff were motivated to provide care. Participants said that they were not able to meet care goals, however, and a DHMT member in Site C’s district identified a, “general feeling that we think that we are hopeless, we think the situation is too hard for us to solve.” The individual criticized that attitude, but most managers and staff in other districts normalized the assumption that nothing could be done. As a manager in Site B’s district said, “we just wait for a miracle to happen [laughs].” That mind-set was rooted in day-to-day experiences, described above, that chronic scarcity was normal and important aspects of the work were beyond control.

The participants’ expectation that the care environment would not improve contributed to a pervasive sense of dependence on other organizations, and NGOs in particular. Some participants described NGO support positively. A Site B manager explained, “it is effective because they [NGOs] are helping us to cut the costs of things like drugs, because if people are not getting sick, it means we are saving.” Other participants questioned programs’ appropriateness. A manager at Site A said, “[NGOs] have their own primary objectives that mostly run in parallel with ours, and in the end, we fail to achieve the indicators that the district wants to achieve.” Although participants differed in their views about program impacts, when asked how supply or funding shortfalls could be addressed, all pointed to NGOs as the most viable option. This normalization of dependence reinforced the tendency toward maintaining relationships with organizations when issues could not be resolved.

Personnel at the facilities valued taking pragmatic action and the common attitude was that it was necessary to make compromises to obtain resources to work with or to make-do with substitutes. As a DHMT member in Site B’s district explained, “we are not in denial that resources are limited at this time. So, we have to use to the maximum those few resources that we have.” These attitudes normalized finding ways to circumvent procedures. Staff who improvised supplies from available materials, or used potentially unsterile equipment, knew they were not following quality-of-care protocols, but justified those actions as a matter of necessity. As a participant at Site C said, “we do that because there is no other option.”

This sense that departures from care standards were unavoidable contributed to demoralization in the facilities we studied. Participants commented that they knew they were not meeting care goals and managers expressed frustration and disappointment about the choices they felt they had to make. A DHMT member in Site A’s district told us,

I know that people are working very hard and I am not happy that there is one nurse [on duty]. Sometimes there is one working, who cannot even go home. They can work for 24-hours, but sometimes there is nothing I can do about it.

Managers knew they would be held accountable by superiors for shortcomings at the facilities and most valued keeping tight control over things they were responsible for. From that perspective, subordinates who questioned decisions were unwelcome, rather than being viewed as potential sources of improvement. We saw this dynamic during a maternal death audit, where senior managers consistently overruled input from staff members and resisted making changes that might imply they had made diagnostic or treatment errors.

Although care providers assumed that people depended on each other, and they valued good working relationships, in these resource-constrained facilities co-workers were often unable to meet each other’s expectations. Care providers consequently became intolerant of those who failed to return support, or did not carry out their duties, such as the nurse noted above who planned to retaliate against her colleague who missed a shift. Responses to resource constraints also influenced attitudes toward leadership. Staff members described additional responsibilities allocated by supervisors as burdens that were avoided when possible. For example, one participant explained that nobody volunteered to be a Quality Improvement Coordinator because, “most people find it hard to work without enough resources to meet their goals, which makes them look like failures.” Demoralization could become severe enough that financial incentives were ineffective. A DHMT member in Site B’s district explained that, “people become frustrated and they just work their [assigned shift] hours and say, ‘no, I am going. I will not work extra hours. The locum which you use, I don’t want it.’” Staff recognized that supervisors could only exert limited control and sometimes used that to their advantage. Participants at Sites A and B described long-standing problems with clinical officers who were frequently absent. The clinical officers realized they could not be easily replaced and we were told that they ignored verbal and written warnings, and some even threatened supervisors with violence in retaliation for disciplinary measures.

### Organizational Cultures and Quality of Care

The enculturated attitudes and behavioral norms we describe above had consequences for care provision in Sites A, B, and C. Some of the ways they weakened quality of care were straightforward. When staff ran out of sterilized equipment, willingness to improvise led to using unsterilized gowns or surgical tools while treating patients. Similarly, normalizing reliance on volunteer blood donations contributed to increased use of relatively high-risk transfusions. Managers and staff also told us that facilities where tasks were shifted to junior cadres misdiagnosed complications or made treatment errors more frequently than other facilities. For example,

[a nurse] acknowledged that there are a lot of problems at this particular health center. In some cases, only one midwife and one medical assistant manned the health center and when they go for a training or workshop, it’s the hospital attendants who do the deliveries. (Field Notes, Site A)

Normalizing such responses to scarcity increased the likelihood of iatrogenic infections and treatment errors.

Other consequences for quality of care involved feedback between assumptions, norms, and constraints. For example, although allocating resources to high-priority activities was reasonable under the circumstances, it meant that managers often did not direct resources to long-term concerns, such as maintenance. That created self-fulfilling conditions, where conserving resources by waiting until equipment malfunctioned, increased the likelihood of severe failures. We noted that at all three sites buildings and important equipment, such as ambulances, were often damaged or non-functional. Correcting the backlog of unaddressed issues became increasingly expensive, contributing to budget shortfalls and reliance on credit, which compounded funding scarcity by requiring that income be used for debt servicing. A similar cycle could occur within wards when care providers focused on patients in obvious distress, or left their assigned positions to assist their colleagues. Routine upkeep tasks, such as cleaning or preparing delivery kits, were left incomplete, which contributed to treatment delays, or to unsterile conditions in the wards.

## Discussion

Raven et al. (2012) define quality of care as, “the performance of interventions according to standards that are known to be safe, which are affordable to the society and that have the ability to produce an impact on mortality, morbidity and disability” (p. 679). Our analysis revealed several points where organizational cultures at the three study sites weakened quality of care. The attitude that “making do” was normal justified using ad hoc solutions which departed from quality assurance standards. Reluctance to expend hard-to-replace resources justified rationing, and targeted allocation of resources contributed to unmet patient care needs and service failures. Another recent study in Malawi also noted that resource constraints contributed to mechanistic, rather than patient-centered care provision (de Kok et al., 2020). Although such practices were preferable to not providing care at all, normalizing them could contribute to mistakes, exposure to infections, delays, and, ultimately, deaths.

The dominant understanding of culture in public health tacitly assumes that it causes behavior (Scott et al., 2003). Following that perspective, an analysis of Sites A, B, and C would identify care providers’ lack of committed and positive leadership, poor teamwork, lack of proactive behavior, and the belief that change was not possible as traits inconsistent with a culture of quality and would recommend training (Raven et al., 2011; Whittaker, 1999) or working with organizational leaders (Brown, 1995; Heiby, 2014; Raven et al., 2011, 2012) to correct those deficiencies. From a perspective informed by Barth’s transactional model of culture, such analyses are not entirely wrong—culture *does* influence behavior when institutionalized cultural elements channel individuals’ actions (Barth, 1966, 1989, 1993; Jenkins, 2008) and other studies have noted ways that aspects of culture, such as organizational hierarchies and gendered experiences, influence care practices (de Kok et al., 2020; Sripad et al., 2018). An analysis following the managerial understanding of culture would be incomplete, however, and describing the culture’s nature only addresses one of Scott et al.’s (2003) points to consider about culture change.

The transactional model suggests that people continually evaluate interactions they are involved in, and cultural traits are maintained or abandoned depending on whether or not outcomes for individuals are adequate within the setting (Barth, 1966, 1989; Jenkins, 2008). This perspective allows us to address all of Scott et al.’s (2003) points. The nature of the cultures in the three Ministry of Health facilities is described above. The cultures were sustained through the day-to-day practices of managers and care providers as they attempted to mitigate problems and limit their exposure to consequences when mishaps occurred. Important resources were scarce or prone to fluctuation, and the Ministry of Health, and the government more generally, was concerned with allocating what was available and maintaining accountability for resource use. The organizational cultures in the facilities fit closely within that wider context.

The remaining point Scott et al. (2003) highlight is the need to understand the culture change processes at work, and in the case of these Ministry of Health facilities, how a culture that would sustain improved quality of care could be fostered. Viewed through a transactional theory lens, constraints on interaction between individuals are primary drivers in culture formation and must also be central to culture change. Trusting subordinates was contrary to existing organizational cultures in Sites A and B, where supervisors assumed that close control over staff was necessary. Successful quality improvement in the United Kingdom, and elsewhere, however, is based on care providers exercising authority and feeling secure about talking through failures (Dekker, 2008; de Kok et al., 2020; Raven et al., 2011; Sripad et al., 2018). Care providers at Site C responded positively to their DHO and DMO’s more open management approach and structural changes delegating authority to lower cadres, and providing consistently safe opportunities for feedback (Dekker, 2008), could promote similar attitudes in other districts.

Members of organizations are also more motivated when their environments are conducive to success (Dekker, 2008; de Kok et al., 2020; Julien et al., 2021; Mathauer & Imhoff, 2006; Sripad et al., 2018; Tancred et al., 2017). Again, Site C illustrates this. It had adequate staffing, reliable water, proportionally larger budget allocations compared to the other sites, and care providers there responded by expressing a sense of self-efficacy and demonstrating effective teamwork. Although Malawi’s government has very limited funds (de Kok et al., 2020; World Bank, 2019), some measures could potentially give MoH personnel more resources to work with and alleviate their sense of chronic scarcity. District Councils could allocate locally raised revenues to health services. Large amounts of NGO funding also already circulate in districts and a larger proportion of existing budgets could be used, as some NGOs already do, to provide material support for health services.

Scott et al. (2003) note the need to be aware of dysfunctional consequences of culture change. In these facilities neither of the options above for increasing district-level health resources is ideal. Councils in districts with strong economies are better positioned than those with weaker ones, such as Site A’s district, to locally fund care. NGO projects are also distributed unevenly, in accordance with donor priorities (Adhikari et al., 2019; Nunnenkamp et al., 2016). These differences could compound health inequities between districts (Marmot et al., 2008) and increased material or funding support from NGOs could reinforce the sense of dependency that already exists among Malawi’s care providers (de Kok et al., 2020).

Cultural elements in the studied facilities emerged from multiple constraints and a combination of measures would be necessary to promote more quality-focused occupational cultures in them; the key requirement is that new policies change existing structures in ways that give district-level personnel greater control over their work, or help to alleviate scarcity and erratic supplies. While failure to address the structural drivers impeding provision of high-quality services leads to new initiatives becoming a source of frustration (de Kok et al., 2020), our findings also suggest that interventions which reduce structural constraints on care provision provide a basis for both improved quality of care and the development of cultural traits supporting it. Such changes encourage taking more action and build self-efficacy, key features of the culture of quality defined in public health literature (Bouchet et al., 2002; Brown, 1995; Raven et al., 2011).

While these findings point to potential avenues for culture change, it is important to keep in mind this study’s limitations. These data were generated over a relatively short period of time, and impacts of culture change take several years, up to a decade, to fully manifest (Bouchet et al., 2002; Curry et al., 2018). The data are also from only three facilities and are not based on standardized instruments that would allow systematic comparison with other facilities in Malawi. It remains, therefore, an open question how widespread the organizational cultures we describe are, and the findings presented here are facility-level cases which should not be taken to represent Malawi’s MoH as a whole. The conditions in the facilities described here are not unique within Malawi (de Kok et al., 2020), or sub-Saharan Africa (Julien et al., 2021; Sripad et al., 2018; Tancred et al., 2017), however, and it may be worth expanding future studies to generate data suitable for investigating organizational culture formation and change processes.

## Conclusion

Viewed from the perspective of transactional theory, culture change occurs perpetually as group members adjust their behaviors in response to new conditions, and experimentation by individuals reveals more attractive strategies, generating new norms, values, and assumptions about how things work (Barth, 1966, 1989, 1993; Jenkins, 2008). Deliberately changing a culture as a means to promote specific policy goals is difficult, because cultural traits are generated in response to multiple contextual factors. There is no single intervention which will reliably generate a culture that policy makers prefer.

The difficulties involved in deliberately creating a culture of quality do not imply that researching culture is unimportant for public health. Structural changes necessary for promoting cultures of quality also improve patient care and may be less costly than maintaining the status quo. In the studied facilities, costs were being transferred informally to patients and their families, services were being reduced when facilities could not fill staffing rosters or lacked essential supplies, and reduced effort by overwhelmed staff impeded care. Day-to-day improvisation and rationing generated opportunity costs when ineffective or incomplete treatment expended resources without producing sustained health improvement. Cultural research based on clearly stated causal theories can assist policy formation that will provide the basis for both improved service quality and the growth of self-sustaining cultures of quality.
